# Regulation and Clinical Implication of Arginine Vasopressin in Patients with Severe Aortic Stenosis Referred to Trans-Catheter Aortic Valve Implantation

**DOI:** 10.3390/medicina56040165

**Published:** 2020-04-06

**Authors:** Hiroyuki Kuwahara, Teruhiko Imamura, Mitsuo Sobajima, Hiroshi Ueno, Koichiro Kinugawa

**Affiliations:** Second Department of Internal Medicine, University of Toyama, Toyama 930-0194, Japan; kuwa19hiro@gmail.com (H.K.); soba1126@yahoo.co.jp (M.S.); hueno@med.u-toyama.ac.jp (H.U.); kinugawa-tky@umin.ac.jp (K.K.)

**Keywords:** hemodynamics, osmolality, heart failure

## Abstract

*Background and objectives*: Plasma arginine vasopressin (P-AVP) is regulated by the non-osmotic pathway in patients with heart failure (HF) and reduced ejection fraction. However, the regulation of P-AVP in patients with severe aortic stenosis (AS) remains unknown. *Materials and Methods*: Consecutive patients with severe AS who received trans-catheter aortic valve implantation (TAVI) between Apr 2016 and Apr 2019 were enrolled in this prospective study. Clinical data including P-AVP were obtained just before TAVI, and the correlation between P-AVP and other variables was investigated. *Results*: In total, 159 patients with severe AS (85.3 ± 4.6 years, male 26%) were enrolled. P-AVP was 1.45 ± 1.13 ng/mL and cardiac index was relatively preserved (2.76 ± 0.54 L/min/m^2^). There was no significant correlation between cardiac index and P-AVP (*p* > 0.05), whereas plasma osmolality had a moderate positive correlation with P-AVP (r = 0.35, *p* < 0.01), predominantly due to blood urea nitrogen (r = 0.27, *p* < 0.01). Patients with diuretics had significantly higher P-AVP than those without diuretics (1.65 ± 1.43 vs. 1.22 ± 0.57 pg/mL, *p* < 0.01). Two-year survivals free from HF readmission were statistically comparable irrespective of the level of pre-procedural P-AVP (*p* = 0.44). *Conclusion*: In patients with severe high-gradient AS who received TAVI, the P-AVP level was dominantly regulated by plasma osmolality instead of arterial underfilling. The clinical implication of elevated P-AVP in the TAVI candidates is the next concern.

## 1. Introduction

Arginine vasopressin (AVP) is a central hormone to maintain homeostasis, regulating water and osmolality balance [1]. AVP is secreted in response to an increase in plasma osmolality, which is monitored by osmoreceptors located in the hypothalamus (osmotic pathway) [2]. Another regulation is a non-osmotic pathway, in which arterial underfilling detected by baroreceptors triggers AVP secretion. 

One of the major receptors that AVP binds to is V2, which is located on the collecting duct in the kidney. When AVP stimulates the V2 receptor, free water is reabsorbed via aquaporin-2, followed by concentrated urine and diluted plasma [3].

In a healthy population, AVP secretion is relatively suppressed and is triggered by increased plasma osmolality (osmotic pathway) [4]. On the contrary, AVP is inappropriately elevated in patients with advanced heart failure [5,6]. Low cardiac output stimulates AVP secretion via a non-osmotic pathway, resulting in diluted hyponatremia due to inappropriate reabsorption of free water in the collecting duct [7]. 

Given the recent introduction of less invasive trans-catheter aortic valve implantation (TAVI) with non-inferior outcomes with the surgery [8], the physiology of elderly patients with severe aortic valve stenosis (AS) who do not indicate surgical aortic valve replacement for their high risk is receiving great concern thus far. Nevertheless, less is known about the regulation of AVP secretion in such a population [9,10]. Detailed knowledge of their AVP regulation would help risk stratification and improve peri-procedural management. 

In this study, we investigated the association of AVP with other clinical parameters to clarify the regulation of AVP in patients with severe AS, as well as the impact of pre-procedural plasma AVP (P-AVP) level on post-TAVI outcomes. 

## 2. Methods

### 2.1. Patient Selection

Consecutive patients who underwent TAVI between Apr 2016 and Apr 2019 for their symptomatic severe AS, defined as aortic valve area <1.0 cm^2^, were prospectively enrolled [11]. Candidates were selected by the heart team, consisting of cardiologists, thoracic surgeons, anesthesiologists, and other clinicians associated with TAVI, according to the indications of the PARTNER trials [12,13]. Patients who received vasopressin type 2 receptor antagonist were excluded given its impact on the AVP level. 

All procedures were performed with a balloon-expandable valve (Sapien XT or Sapien 3; Edwards Lifesciences Inc.) or self-expandable valve (CoreValve or Evolut R or Evolut PRO; Medtronic Inc) via trans-femoral approach or alternative approach under general or local anesthesia. Following the procedure, all patients received guideline-directed medical therapy. Informed consent was obtained beforehand, and this study was approved by the local institutional review board on Aug 13, 2018 (IRB 30-416). 

### 2.2. Data Collection

General hemodynamic data were obtained by right heart catheterization within one month before the procedure. Standard echocardiographic data were also obtained within one month before the procedure. The aortic valve area was calculated by a continuity equation [14]. Within one week before the procedure, laboratory data including P-AVP and urine data including aquaporin-2 were obtained. 

Following TAVI, the heart failure readmission that required IV diuretics under careful in-hospital observation and all-cause death were counted during the two-year observational period. Within two weeks following TAVI, the above-described clinical data were obtained. 

### 2.3. Statistical Analyses

Statistics were performed with JMP pro ver14.2 (SAS Institute Inc). Two-sided *p*-values < 0.05 were considered statistically significant. Continuous variables were expressed as mean and standard deviation and compared between the groups by unpaired *t*-test or Mann–Whitney U test considering their distributions. Categorical variables were presented as a number with percentage and were compared by Fisher’s exact test. Correlations of P-AVP with other clinical variables were assessed by Pearson’s correlation coefficient. Clinical variables before and after TAVI were compared by paired *t*-test or Wilcoxon signed-rank test as appropriate. Impact of baseline P-AVP on two-year freedom from death or heart failure readmissions were compared between the two groups stratified by the median P-AVP levels by the log-rank test.

## 3. Results

### 3.1. Baseline Characteristics

In total, 155 patients with severe AS who received TAVI were enrolled. Baseline characteristics are summarized in Table 1. The mean age was 85.4 ± 4.6 years old, and 38 (24%) were male. All patients had severe AS with aortic valve area 0.58 ± 0.15 cm^2^, mean pressure gradient through aortic valve 50.0 ± 17.2 mmHg, and peal velocity of aortic valve flow 4.51 ± 0.73 m/sec.

Left ventricular diastolic diameter was 45.8 ± 6.6 mm and ejection fraction was 62.9% ± 11.7%. Hemodynamics were relatively preserved, with right atrial pressure of 5.4 ± 2.7 mmHg, pulmonary artery wedge pressure of 12.3 ± 5.0 mmHg, and cardiac index of 2.76 ± 0.54 L/min/m^2^. Urine osmolality and aquaporin-2 concentration were also relatively preserved. 

Society of Thoracic Surgeons score was 6.7% ± 3.4%, and most of the patients (96%) had heart failure symptoms with New York Heart Association functional class II or III, and plasma B-type natriuretic peptide level was 377.6 ± 370.0 pg/mL.

### 3.2. Measurement of P-AVP

**Figure 1:**
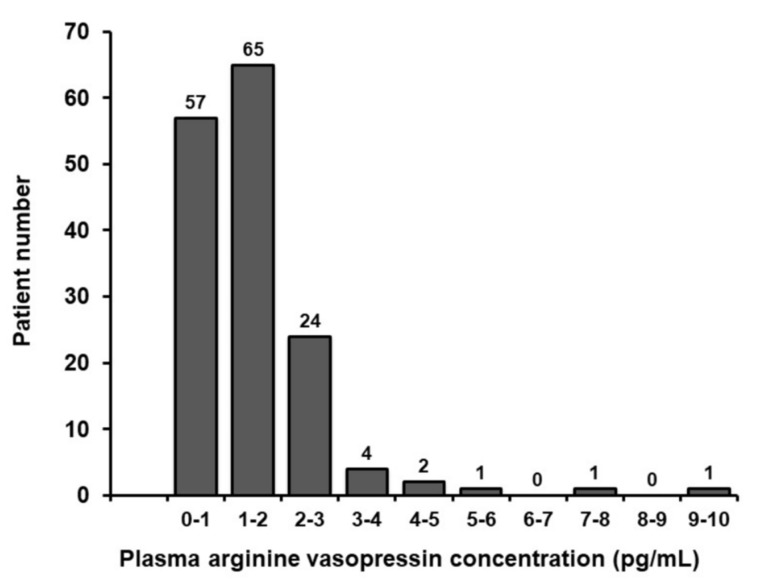
Distribution of plasma arginine vasopressin concentration.

P-AVP averaged 1.45 ± 1.13 pg/mL. The distribution of P-AVP is shown in Figure 1. Most patients (94%) had P-AVP between 0.0 and 3.0 pg/mL, whereas others had relatively high values between 3.0 and 10.0 pg/mL.

### 3.3. Association of P-AVP with Other Baseline Clinical Parameters

P-AVP had no statistically significant correlations with hemodynamic parameters, including right atrial pressure and cardiac index (*p* > 0.05 for all; Table 2). There were also no significant associations between P-AVP level and the parameters associating with the severity of AS, as well as left atrial diameter (*p* > 0.05 for all). In a sub-analysis of the patients with cardiac index below 2.2 L/min/m^2^ (N = 19), there was again no significant association between P-AVP level and cardiac index (*p* = 0.74). 

On the contrary, P-AVP had a statistically significant correlation with plasma osmolality (r = 0.35, *p* < 0.01), dominantly due to blood urea nitrogen (r = 0.27, *p* < 0.01), instead of sodium and glucose (*p* > 0.05 for both) (Table 3). Elevated P-AVP was associated with increased excretion of aquaporin-2 in urine, as expected (r = 0.48, *p* < 0.01).

**Figure 2:**
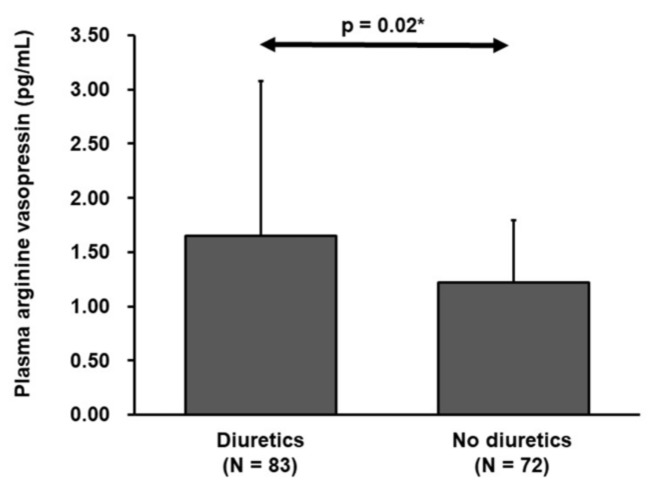
Plasma arginine vasopressin concentration stratified by the use of diuretics. * *p* < 0.05 by unpaired *t*-test.

Eighty-three patients received diuretics, whereas 72 did not. Patients with diuretics had significantly higher P-AVP compared to those without diuretics (1.65 ± 1.43 vs. 1.22 ± 0.57 pg/mL, *p* = 0.02; Figure 2).

### 3.4. Clinical Variables Following TAVI

Echocardiographic parameters associated with the severity of AS, as well as B-type natriuretic peptide levels, improved significantly following TAVI (Appendix A; *p* < 0.05). P-AVP decreased statistically significantly from 1.45 ± 1.13 to 1.29 ± 0.93 pg/mL (*p* < 0.01), although the absolute value was clinically trivial. Similarly to the pre-TAVI analyses, post-TAVI P-AVP had a statistically significant correlation with plasma osmolality (N = 150; r = 0.41, *p* < 0.01) but had no significant correlation with cardiac index (N = 54; r = -0.05, *p* = 0.71). 

### 3.5. Prognostic Implication of P-AVP Following TAVI

**Figure 3:**
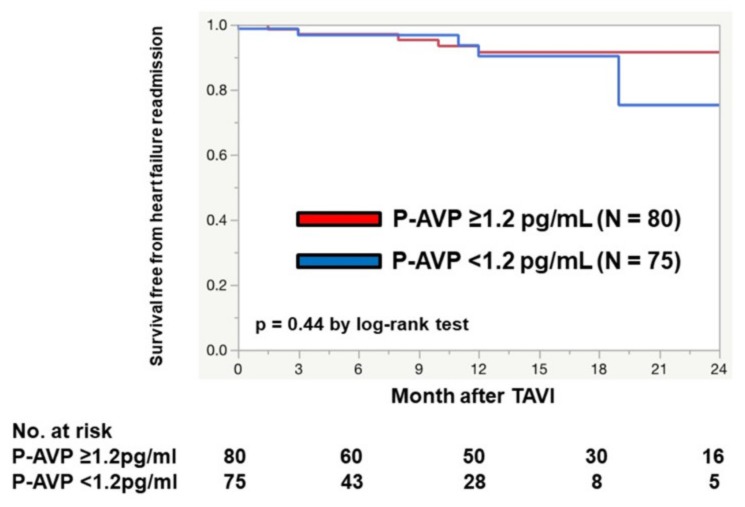
Survival free from heart failure readmission stratified by the plasma level of arginine vasopressin.

During the two-year follow-up period following TAVI, 10 patients died (5 cardiovascular events and others including pulmonary pneumonia, malignancy, and sudden death) and 9 experienced heart failure readmissions. Patients were stratified into two groups by the median baseline P-AVP of 1.20 pg/mL. Survival free from heart failure readmission was not statistically stratified by the median baseline P-AVP (88.5% vs. 92.3%, *p* = 0.44; Figure 3). Post-TAVI median P-AVP (1.0 pg/mL) also did not stratify patients’ survival free from heart failure readmissions (*p* = 0.75). Post-TAVI use of diuretics at index discharge (39%) also did not have any prognostic impact (*p* = 0.43). 

## 4. Discussion

In this study, we investigated the association of P-AVP with other clinical data among the patients with severe AS receiving TAVI as well as the prognostic impact of baseline P-AVP on post-TAVI outcome. Major findings are the following: (1) AVP secretion was relatively suppressed in patients with severe AS; (2) peri-procedural P-AVP had no significant association with severity of heart failure or AS (non-osmotic pathway) but was associated with plasma osmolality dominantly due to blood urea nitrogen (osmotic pathway); (3) peri-procedural P-AVP had no significant impact on post-TAVI outcome. 

### 4.1. Regulation of p-AVP: Non-Osmotic Pathway vs. Osmotic Pathway

In general, P-AVP is inappropriately elevated in heart failure patients despite low plasma osmolality [15]. P-AVP has a positive correlation with the severity of heart failure [3]. In particular, low cardiac output triggers huge excretion of AVP via the non-osmotic pathway [7], which stimulates reabsorption of free water in the collecting duct and facilitates water retention [6]. 

In our cohort, despite relatively worse heart failure symptoms and elevated intra-cardiac load, indicating severe heart failure, P-AVP was relatively lower and “appropriate” for their normal plasma osmolality. Despite the severe heart failure they had as symptoms, P-AVP had no association with hemodynamics (non-osmotic pathway) but instead with plasma osmolality (osmotic pathway). 

The major reason why P-AVP was regulated by the osmotic pathway would come from the relatively preserved cardiac output in our cohort (84% had cardiac index above 2.2 L/min/m^2^). Following TAVI, although statistically significant, the absolute value of changes in P-AVP was trivial. Post-TAVI P-AVP was consistently correlated with plasma osmolality instead of cardiac output. Given that hemodynamics was already well-preserved before TAVI, TAVI might not have any significant impact on P-AVP. 

Currently, it is highly recommended to perform TAVI before deterioration of cardiac function, given the evidence that post-TAVI outcome is worse in patients with low-flow, low-gradient AS [16]. Although the patients with such a too-progressed AS are not good candidates for TAVI and are rarely referred to TAVI in the current literature, their P-AVP might be regulated differently from our cohort. 

We also found that the use of diuretics was associated with elevated P-AVP. This is compatible with the previous animal experiment [17], in which P-AVP increased following the administration of furosemide. Volume reduction by diuretics might increase plasma osmolality and trigger the secretion of AVP. The implication of increased P-AVP following the administration of diuretics remains a future concern.

Overall, excretion of aquaporin-2 in urine in response to AVP was preserved. We did not include those receiving tolvaptan, a vasopressin type-2 receptor antagonist, but there might be a certain number of responders to tolvaptan in our cohort. It is unknown whether responses to tolvaptan in the AS cohort can be explained in the same manner as the general heart failure cohort [18]. 

### 4.2. Impact of P-AVP on Post-TAVI Outcome

In patients with heart failure with reduced ejection fraction, the EVEREST trial showed that P-AVP above 8 pg/mL was associated with higher mortality [19]. Our team showed in patients with advanced heart failure that P-AVP above 5.3 pg/mL was associated with cardiac death [7]. 

Baseline P-AVP had no significant impact on post-TAVI survival free from heart failure readmission in our cohort. Even though they had severe heart failure symptoms, their cardiac output was relatively preserved. As a result, P-AVP was not an index of the severity of heart failure and AS, which might be one reason why baseline P-AVP had no prognostic impact in our cohort. Another explanation would be relatively low P-AVP in our cohort (mean value 1.45 pg/mL) compared with other studies. The implication of P-AVP among those with more elevated P-AVP, which might be observed in those with low-flow and low-gradient AS, remain a future concern. The changes in P-AVP following TAVI and its clinical implications are also future concerns. 

### 4.3. Study Limitations

The sample size is moderate, but we collected comprehensive clinical data, although further data including frailty would have improved our findings. Our findings should be validated in larger-scale studies. Our study lacked the longitudinal follow-up of P-AVP. 

We should pay special attention that our findings can be adopted only for those with severe symptomatic aortic valve stenosis, mostly left ventricular hypertrophy, and also mostly preserved ejection fraction. Our findings cannot simply be adopted into those with reduced ejection fraction (i.e., low-flow log-gradient aortic valve stenosis), mild aortic valve stenosis, and left ventricular hypertrophy due to other etiologies. Furthermore, the association between arginine vasopressin and plasma osmolality following surgical aortic valve replacement remains unknown.

P-AVP did not have prognostic implications on post-TAVI mortality and heart failure readmission, but the impact of P-AVP on other clinical outcomes, including exercise capacity, quality of life, and other comorbidities, remains a future concern. In such studies, other clinically significant factors should be adjusted for.

## 5. Conclusions

In elderly patients with severe AS receiving TAVI, cardiac output was relatively preserved, the P-AVP level was not associated with the severity of AS and post-TAVI clinical outcomes, and P-AVP might not be a good marker for them. The implication of elevated P-AVP and any intervention for the elevated P-AVP in the TAVI candidates are the next concerns.

## Figures and Tables

**Table 1:** Baseline characteristics.

	N = 155
**Demographics**	
Age (years)	85.4 ± 4.6
Male	38 (24%)
Body surface area (m^2^)	1.40 ± 0.17
NYHA functional class	I (3), II (75), III (74), IV (3)
STS score (%)	6.7 ± 3.4
Hypertension	115 (74%)
Diabetes mellitus	24 (15%)
**Medication**	
ACE inhibitor/ARB	87 (56%)
Beta-blocker	49 (32%)
Aldosterone antagonist	43 (28%)
Diuretics	83 (54%)
**Laboratory data**	
Blood urea nitrogen (mg/dL)	23.9 ± 9.8
Creatinine (mg/dL)	1.0 ± 0.4
Sodium (mEq/L)	140.2 ± 2.5
Glucose (mg/dL)	113.3 ± 35.2
B-type natriuretic peptide (pg/mL)	377.6 ± 370.0
Plasma osmolality (mOsm/L)	290.1 ± 6.3
Plasma arginine vasopressin (pg/mL)	1.45 ± 1.13
**Urine data**	
Urine osmolality (mOsm/L)	435.4 ± 123.0
Urine sodium (mEq/L)	80.2 ± 29.6
Urine creatinine (mg/dL)	67.6 ± 37.6
Urine aquaporin-2 (ng/mL)	3.35 ± 4.00
**Echocardiogram**	
Left ventricular diastolic diameter (mm)	45.8 ± 6.6
Left ventricular ejection fraction (%)	62.9 ± 11.7
Left atrial diameter, mm	42 ± 9
Peak velocity of aortic valve flow (m/sec)	4.51 ± 0.73
Mean pressure gradient through aortic valve (mmHg)	50.0 ± 17.2
Aortic valve area (cm^2^)	0.58 ± 0.15
**Hemodynamics**	
Right atrial pressure (mmHg)	5.4 ± 2.7
Mean pulmonary artery pressure (mmHg)	19.6 ± 5.6
Pulmonary artery wedge pressure (mmHg)	12.3 ± 5.0
Cardiac index (L/min/m^2^)	2.76 ± 0.54

NYHA, New York Heart Association; STS, Society of Thoracic Surgeons. Variables are expressed as mean and standard deviation or number and percentage.

**Table 2:** Correlation of plasma arginine vasopressin level with hemodynamics and severity of aortic valve stenosis.

	r Value	*p*-Value
**Hemodynamics**		
Right atrial pressure (mmHg)	−0.06	0.41
Pulmonary artery wedge pressure (mmHg)	0.00	0.98
Cardiac index (L/min/m^2^)	−0.13	0.10
**Echocardiography data**		
Left atrial diameter (mm)	0.12	0.32
Peak velocity (m/s)	−0.02	0.84
Mean pressure gradient (mmHg)	0.01	0.94
Aortic valve area (cm^2^)	−0.02	0.84
**Laboratory data**		
B-type natriuretic peptide (pg/mL)	0.13	0.12

Associations were investigated by Pearson’s correlation coefficient.

**Table 3:** Correlation of plasma arginine vasopressin level with laboratory data.

	r Value	*p*-Value
Plasma osmolality (mOsm/L)	0.35	<0.01 *
Sodium (mEq/L)	0.07	0.36
Blood urea nitrogen (mg/dL)	0.27	<0.01 *
Glucose (mg/dL)	−0.07	0.34
Urine aquaporin-2 (ng/mL)	0.48	<0.01 *

* *p* < 0.05 by Pearson’s correlation coefficient.

## Appendix A

**Table A1:** Clinical parameters before and after TAVI.

	Baseline	After TAVI	*p*-Value
Laboratory data			
Blood urea nitrogen (mg/dL)	23.9 ± 9.8	25.8 ± 9.6	<0.01 *
Creatinine (mg/dL)	1.0 ± 0.4	0.95 ± 0.4	<0.01 *
Sodium (mEq/L)	140.2 ± 2.5	139.1 ± 3.1	<0.01 *
Glucose (mg/dL)	113.3 ± 35.2	98.7 ± 29.2	<0.01 *
B-type natriuretic peptide (pg/mL)	377.6 ± 370.0	174.8 ± 193.3	<0.01 *
plasma osmolality (mOsm/L)	290.1 ± 6.3	288.3 ± 7.3	<0.01 *
Arginine vasopressin (pg/mL)	1.45 ± 1.13	1.29 ± 0.93	<0.01 *
Urine data			
Urine osmolality (mOsm/L)	435.4 ± 123.0	434.6 ± 138.8	0.97
Urine sodium (mEq/L)	80.2 ± 29.6	75.6 ± 27.3	0.06
Urine creatinine (mg/dL)	67.6 ± 37.6	67.4 ± 34.5	0.78
Urine aquaporin-2 (ng/mL)	3.4 ± 4.0	4.2 ± 4.9	0.17
Echocardiogram			
Left ventricular diastolic diameter (mm)	45.8 ± 6.6	45.5 ± 6.3	0.15
Left ventricular ejection fraction (%)	62.9 ± 11.7	65.1 ± 10.7	<0.01 *
Peak velocity of aortic valve flow (m/s)	4.51 ± 0.73	2.10 ± 0.47	<0.01 *
Mean pressure gradient through aortic valve (mmHg)	50.0 ± 17.2	10.3 ± 5.0	<0.01 *
Aortic valve area (cm^2^)	0.58 ± 0.15	1.47 ± 0.30	<0.01 *

Variables were compared between two groups by paired *t*-test or Wilcoxon signed-rank test as appropriate. * *p* < 0.05.

## References

[1] Robertson G.L. (2001). Antidiuretic hormone. Normal and disordered function. Endocrinol. Metab. Clin. N. Am..

[2] Robertson G.L., Mahr E.A., Athar S., Sinha T. (1973). Development and clinical application of a new method for the radioimmunoassay of arginine vasopressin in human plasma. J. Clin. Investig..

[3] Funayama H., Nakamura T., Saito T., Yoshimura A., Saito M., Kawakami M., Ishikawa S.E. (2004). Urinary excretion of aquaporin-2 water channel exaggerated dependent upon vasopressin in congestive heart failure. Kidney Int..

[4] Saito T., Ishikawa S.E., Sasaki S., Nakamura T., Rokkaku K., Kawakami A., Honda K., Marumo F., Saito T. (1997). Urinary excretion of aquaporin-2 in the diagnosis of central diabetes insipidus. J. Clin. Endocrinol. Metab..

[5] Goldsmith S.R., Francis G.S., Cowley A.W., Goldenberg I.F., Cohn J.N. (1986). Hemodynamic effects of infused arginine vasopressin in congestive heart failure. J. Am. Coll. Cardiol..

[6] Szatalowicz V.L., Arnold P.E., Chaimovitz C., Bichet D., Berl T., Schrier R.W. (1981). Radioimmunoassay of plasma arginine vasopressin in hyponatremic patients with congestive heart failure. N. Engl. J. Med..

[7] Imamura T., Kinugawa K., Hatano M., Fujino T., Inaba T., Maki H., Kinoshita O., Nawata K., Kyo S., Ono M. (2014). Low cardiac output stimulates vasopressin release in patients with stage d heart failure. Circ. J..

[8] Mack M.J., Leon M.B., Smith C.R., Miller D.C., Moses J.W., Tuzcu E.M., Webb J.G., Douglas P.S., Anderson W.N., Blackstone E.H. (2015). 5-year outcomes of transcatheter aortic valve replacement compared with standard treatment for patients with inoperable aortic stenosis (PARTNER 1): A randomised controlled trial. Lancet.

[9] Ramirez-Gil J.F., Drobinski G., Carayon A., Isnard R., Hoffman O., Sotirov I., Chanton E., Montalescot G., Thomas D. (1993). [Neurohormonal profile in aortic valve stenosis]. Arch. Mal. Coeur Vaiss..

[10] Jensen L.W., Bagger J.P., Pedersen E.B. (1996). Twenty-four-hour ambulatory blood pressure and vasoactive hormones in valvular aortic disease. Blood Press.

[11] Nishimura R.A., Otto C.M., Bonow R.O., Carabello B.A., Erwin J.P., Guyton R.A., O’Gara P.T., Ruiz C.E., Skubas N.J., Sorajja P. (2014). 2014 AHA/ACC guideline for the management of patients with valvular heart disease: Executive summary: A report of the American College of Cardiology/American Heart Association task force on practice guidelines. Circulation.

[12] Smith C.R., Leon M.B., Mack M.J., Miller D.C., Moses J.W., Svensson L.G., Tuzcu E.M., Webb J.G., Fontana G.P., Makkar R.R. (2011). Transcatheter versus surgical aortic-valve replacement in high-risk patients. N. Engl. J. Med..

[13] Leon M.B., Smith C.R., Mack M., Miller D.C., Moses J.W., Svensson L.G., Tuzcu E.M., Webb J.G., Fontana G.P., Makkar R.R. (2010). Transcatheter aortic-valve implantation for aortic stenosis in patients who cannot undergo surgery. N. Engl. J. Med..

[14] Zoghbi W.A., Farmer K.L., Soto J.G., Nelson J.G., Quinones M.A. (1986). Accurate noninvasive quantification of stenotic aortic valve area by Doppler echocardiography. Circulation.

[15] Schrier R.W., Abraham W.T. (1999). Hormones and hemodynamics in heart failure. N. Engl. J. Med..

[16] Fischer-Rasokat U., Renker M., Liebetrau C., van Linden A., Arsalan M., Weferling M., Rolf A., Doss M., Möllmann H., Walther T. (2019). 1-year survival after TAVR of patients with low-flow, low-gradient and high-gradient aortic valve stenosis in matched study populations. JACC Cardiovasc. Interv..

[17] Miyazaki T., Fujiki H., Yamamura Y., Nakamura S., Mori T. (2007). Tolvaptan, an orally active vasopressin V(2)-receptor antagonist—Pharmacology and clinical trials. Cardiovasc. Drug Rev..

[18] Mitsui M., Kataoka A., Nara Y., Nagura F., Kawashima H., Hioki H., Nakashima M., Watanabe Y., Yokoyama N., Kozuma K. (2019). Clinical safety and efficacy of tolvaptan for acute phase therapy in patients with low-flow and normal-flow severe aortic stenosis. Heart Vessel..

[19] Lanfear D.E., Sabbah H.N., Goldsmith S.R., Greene S.J., Ambrosy A.P., Fought A.J., Kwasny M.J., Swedberg K., Yancy C.W., Konstam M.A. (2013). Association of arginine vasopressin levels with outcomes and the effect of V2 blockade in patients hospitalized for heart failure with reduced ejection fraction: Insights from the EVEREST trial. Circ. Heart Fail..

